# Application of Generalized Finite Difference Method for Nonlinear Analysis of the Electrothermal Micro-Actuator

**DOI:** 10.3390/mi16030325

**Published:** 2025-03-12

**Authors:** Hao Chen, Xiaoyu Kong, Xiangdong Sun, Mengxu Chen, Haiyang Yuan

**Affiliations:** 1 Engineering School, Lishui University, Lishui 323000, China; yuanhaiyang1156@163.com; 2 State Key Laboratory of Precision Manufacturing for Extreme Service Performance, Central South University, Changsha 410083, China; 3 School of Mechanical Engineering, Nanjing University of Science and Technology, Nanjing 210094, China; kxy0022@njust.edu.cn; 4 School of Electrical Engineering, Xi’an University of Technology, Xi’an 710048, China; sxd1030@163.com; 5 Zhenhang Industrial Group Co., Ltd., Lishui 323000, China; boss@greenfilter.cn

**Keywords:** generalized finite difference method, meshless, electrothermal actuator, incremental load method

## Abstract

In this work, the generalized finite difference method (GFDM), a popular meshless numerical method, is employed for predicting the thermal and mechanical behavior of an electrothermal micro-actuator. Based on the concept of GFDM and discretization on the computational domain, the discrete forms of the thermal and mechanical governing equations are derived, respectively. With the help of the incremental load method, the discrete form from the electrothermal analysis is solved precisely and the temperature distribution is obtained. Meanwhile, combining this approach with the discrete control equation derived from the natural boundary condition, its displacement is also evaluated. The convergence of the temperature by different iterative methods is tested and compared. The computational stability and efficiency (CPU time) in these two analyses are also given in this study. To further investigate the accuracy of the solutions, experiments to capture temperature and FEM analysis are conducted. Regardless of the imperfect boundary condition, the temperature distribution calculated by the GFDM shows great agreement with that obtained by experiment and FEM. A similar phenomenon can be also found in the comparison between the displacements evaluated by the GFDM and FEM, respectively.

## 1. Introduction

Playing an important role in micro- and nano-electromechanical systems (MEMS and NEMS), the micro-actuator has a wide spectrum of applications, including the micro-grippers [1,2], precise micro-positioning [3], bistable/multistable micro-switch [4,5], etc. When the micro-actuator is driven in the system, the electrical signal is excited and converted into motion or operation under the interaction of multiple physical fields. Thus, according to the different mechanisms of action, the actuator family can be divided into piezoelectric [6], electrostatic [7], electrothermal [8], electromagnetic [9], and so on. Increasing attention has been given to the electrothermal actuator due to its superior performance in terms of displacement, driving force, and process compatibility.

In recent years, numerous numerical computational approaches, distinct from the classical finite element method (FEM), have been employed to predict the behavior of micro-actuators. Ouakad et al. [10] tried to apply the meshless Galerkin decomposition technique for the equations governing the steady-state behavior of the microbeam, which is electrostatically actuated and initially curved. To investigate the motion of the sandwich symmetric functionally graded plates with piezoelectric layers, the local Petrov–Galerkin method (MLPG), a meshless method, was adopted for the numerical evaluation [11]. Chen et al. [12] implemented the local radial point interpolation method (LRPIM) for the dynamic analysis of the electrothermal structure, in which the results are validated by temperature. In addition, Nguyen et al. [13] explored a novel implementation of a physics-informed neural network (PINN) for predicting the deformation and identifying the material coefficient through inverse analysis of the piezoelectric actuator. In order to simulate the response of piezoelectric devices, Xia et al. [14] applied the meshless generalized finite difference method (GFDM) for the electroelastic analysis of piezoelectric structures. The fast development on micro-manipulation intensifies the demand on the actuating performance. The structural optimization, dependent on the robust numerical method, can be the key to addressing the requirement. Those meshless methods mentioned above remove the Lagrange grid, which usually causes the distortion of the calculation results because of the poor-quality grid and thus the increase in the optimization iteration time. However, it is still a challenge for them to be adopted in the optimization. For the Galerkin decomposition technique and MLPG, they need to construct the global or local background grid to calculate the integral derived from weak form [11,15,16]. Thus, it will be difficult to handle the background grids near the complex boundary due to some areas of the grids not being within the computational domain. Though the PINN, a strong form method, can overcome the shortcoming, its generalization ability on different structural shapes is poorer. This is to say that the neural network needs to be trained continuously, which means a substantial time cost [17,18]. As another strong form meshless method, the GFDM has high computational efficiency [19,20] and has been applied into the topology optimization for heat conduction structure [21]. Nonetheless, the current strategy, based on the GFDM, struggles to deal accurately with high nonlinearity, which will be depicted and compared in the electrothermal analysis of this study.

As a case study for the micro-actuator, the main objective is to develop an efficient and robust convergent numerical scheme based on the GFDM for the electrothermal and thermomechanical analysis of the V-shaped actuator. The structure of this paper is as follows: In Section 2, the fundamental principle of the GFDM and the flowchart for the analysis of the actuator are depicted. Section 3 provides the discretization equation via the combination of the GFDM and incremental loading method, based on the pre-derived electrothermal coupling equation. In Section 4, according to the thermoelastic model, the deflection is estimated via the GFDM. In Section 5, the results of the thermal and mechanical analysis, obtained via GFDM, FEM, and experiment, are presented and compared. The accuracy and convergence of the GFDM is also tested. Finally, Section 6 provides some concluding remarks and suggest future research directions.

## 2. Generalized Finite Difference Method

To facilitate the understanding, a general 2D boundary value problem, solving by the GFDM approach, is derived here [22]. The problem domain Ω in the *xoy* plane is first dispersed into *N* points. For any discrete point (*x_I_*, *y_I_*), the other *N^l^* discrete points are found in its support domain. With Taylor series expansion and field variable *u_I_*, the *u_j_* at the point (*x_j_*, *y_j_*), *j* ∈ *N^l^*, can be expressed as

(1) $$\begin{array}{l} u_{j}=u_{I}+h_{jI}u_{I,x}+k_{jI}u_{I,y}+\frac{1}{2}h_{jI}^{2}u_{I,xx}+\\ \frac{1}{2}k_{jI}^{2}u_{I,yy}+h_{jI}k_{jI}u_{I,xy}+\ldots \end{array}$$

where *h_jI_* = *x_j_* − *x_I_* and *k_jI_* = *y_j_* − *y_I_*_,_ *u_I_*_,*xy*_ denote the second derivative of the field variable *u_I_* versus *x* and *y*. Omitting the third and higher-order derivative terms, the Taylor series expansion of the *N^l^* discrete points by matrix form is

(2) Ax+b=0,

where **A** with *N^l^* × 5 is

(3) $$A={\left[\begin{array}{ccccc} x_{1I} & \cdots & x_{jI} & \cdots & x_{N^{l}I}\\ y_{1I} & \cdots & y_{jI} & \cdots & y_{N^{l}I}\\ x_{1I}^{2}/2 & \cdots & x_{jI}^{2}/2 & \cdots & x_{N^{l}I}^{2}/2\\ y_{1I}^{2}/2 & \cdots & y_{jI}^{2}/2 & \cdots & y_{N^{l}I}^{2}/2\\ x_{1I}y_{1I} & \cdots & x_{jI}y_{jI} & \cdots & x_{N^{l}I}y_{N^{l}I} \end{array}\right]}^{T},$$

***x*** with 5 × 1 is

(4) $$x={\left(\begin{array}{lllll} u_{I,x} & u_{I,y} & u_{I,xx} & u_{I,yy} & u_{I,xy} \end{array}\right)}^{T},$$

and ***b*** with *N^l^* × 1 is

(5) $$b={\left(\begin{array}{lllll} u_{I}-u_{1} & \ldots & u_{I}-u_{j} & \ldots & u_{I}-u_{N^{l}} \end{array}\right)}^{T}.$$

Thus, a local error function *F* at the point (*x_I_*, *y_I_*) is defined as

(6) $$F(x)={\left(Ax+b\right)}^{T}W(Ax+b),$$

where

(7) $$W=diag(w_{1I},\ldots w_{jI},\ldots w_{N^{l}I}).$$

The *w_jI_* is obtained by the weight function *w*(*d*).

(8) $$w(r)=\left\{\begin{array}{c} 1-6r^{2}+8r^{3}-3r^{4},r\leq 1\\ 0,\;r>1 \end{array}\right.$$

where radius *r*, *d*/*d*_m_, and *d* represents the distance between point (*x_I_*, *y_I_*) and (*x_j_*, *y_j_*). To minimize the value of the local error function, we have

(9) $$\frac{\partial F(x)}{\partial x}=2A^{T}W(Ax+b)=0.$$

Further, we obtain

(10) $$x={\left(A^{T}WA\right)}^{-1}A^{T}Wb.$$

To facilitate the final matrix assembly, vector ***b*** can be rewritten as

(11) b=Ea,

where matrix ***E*** with *N^l^* × (*N^l^* + 1) is as follows:

$$E=\left[\begin{array}{ccccc} -1 & 1 & & & \\ -1 & & 1 & & \\ \vdots & & & \ddots & \\ -1 & & & & 1 \end{array}\right],$$

and ***a*** with (*N^l^* + 1) ×1 is as follows:

(12) $$\alpha ={\left(u_{I},u_{1},\ldots u_{j},\ldots u_{N^{l}}\right)}^{T}.$$

Thus, the shape function matrix **N** is as follows:

(13) $$N={\left(A^{T}WA\right)}^{-1}A^{T}WE,$$

and Equation (10) can be rewritten as

(14) x=Na.

**Figure 1:**
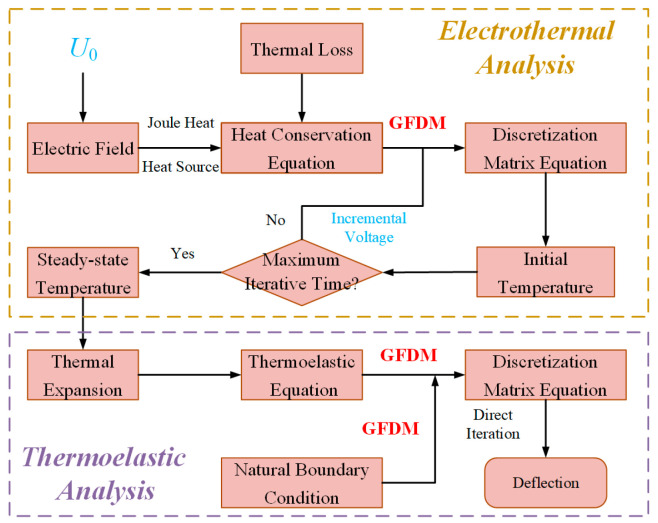
Flowchart using GFDM to evaluate the thermal and mechanic behavior of the actuator.

In the 2D boundary value problem, the derivative terms either in the PDE or in the boundary condition can be all replaced by the linear equation in (14). Based on the GFDM, the flowchart used to evaluate the thermal and mechanical behavior of the actuator is presented in Figure 1.

## 3. Electrothermal Analysis

**Figure 2:**
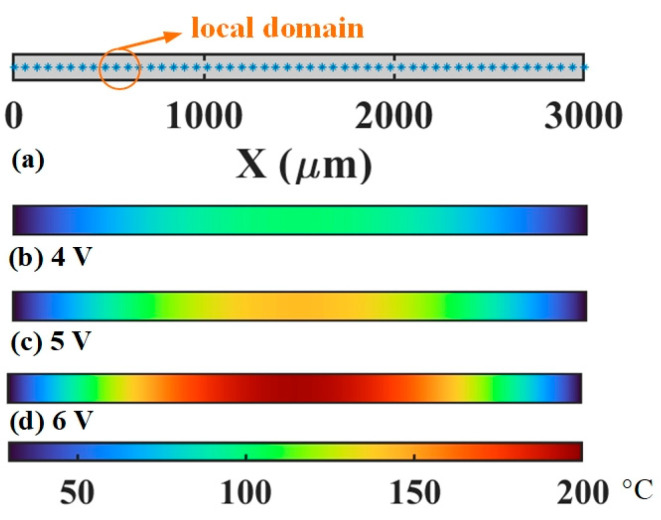
One-dimensional computational model and temperature distribution of the actuator in the electrothermal analysis. (**a**) Computational model. (**b**) Temperature at 4 V. (**c**) Temperature at 5 V. (**d**) Temperature at 6 V.

A nearly identical cross-section can be found in the V-shaped actuator fabricated with polysilicon. Thus, according to our previous study [23,24], a simplified 1D geometric model is established as shown in Figure 2 for the electrothermal analysis. Here, for any infinitesimal element d*x* in the model, its heat conversation equation constitutes heat loss and heat generation terms. The heat conduction, due to the temperature difference between the d*x* and its two adjacent elements, is considered in the heat loss. Furthermore, the heat fluxes from d*x* to the air and the substrate are counted as the heat loss. The heat generation source arises from the Joule effect under the electric filed. Therefore, the coupling equation of the actuator between the electric and thermal field can be presented as

(15) $$k(T)\frac{\partial^{2}T}{\partial x^{2}}-(k_{v}\frac{w+2h}{wh}+\frac{S}{h})T+\frac{U_{0}^{2}\rho (T)}{(\int_{0}^{2l}\rho (T)dx{)}^{2}}\;=\;0,$$

and the boundary condition and initial condition are

(16) T(0)=0,T(2l)=0,

where *k* and *ρ* denote the thermal conductivity and electrical resistivity changing with the temperature; *k*_v_ and *S* are the dissipation rate of heat separately flowing to the air and substrate by air gap; and *U*_0_ is the voltage applied on the actuator. According to the GFDM above, in Equation (14) $x={\left(T_{I,x},T_{I,xx}\right)}^{T}$, $a={\left(T_{I},T_{1},\ldots T_{j},\ldots T_{N^{l}}\right)}^{T}$. For the shape function matrix **N** in Equation (13),

(17) $$A={\left[\begin{array}{ccccc} x_{1I} & \cdots & x_{jI} & \cdots & x_{N^{l}I}\\ x_{1I}^{2}/2 & \cdots & x_{jI}^{2}/2 & \cdots & x_{N^{l}I}^{2}/2 \end{array}\right]}^{T}.$$

When the vector **x** is plugged in the Equation (15), the discretization form at point *x_I_* can be rewritten as

(18) $$K_{I}a=p_{I},$$

where

(19) $$K_{I,1}=k(T_{I})*N_{2,1}-(k_{v}\frac{w+2h}{wh}+\frac{S}{h}),$$

(20) $$K_{I,j}=k(T_{I})*N_{2,j},\;j=2,3,\ldots (N^{l}+1),$$

and

(21) $$p_{I}=-U_{0}^{2}\rho (T_{I})/(\frac{2l}{N}\sum_{j=1}^{N-1}\rho (T_{j})).$$

Finally, the single Equation (18) needs to be assembled into the total matrix Equation (22):

(22) Ka=P,

where the matrix **K** is with *N* × *N* and vector **P** is with *N* × 1. For the element $K_{I,j}$, *I* is the global index of the point *x_I_*, and *j* is the local index of the point *x_j_*. The global index of the *j* is found and defined as *J*. Thus, the element $K_{I,j}$ should be placed in the *I*th row and *J*th column of the total stiffness matrix **K**. The *p_I_* is located in the *I*th row of the **P**. After the assembling, the first row and last row in the **K** and **P** should be rewritten to meet the Dirichlet boundary condition. Given the high nonlinearity existing in the material parameters, the incremental load method should be applied to the iteration process for solving Equation (22). For each iterative step, the voltage will be $\sqrt{U_{0}^{2}/N^{U}}$ larger than the last step, where *N^U^* is the iteration times. This generates a linear increasing on the load vector **P**. The detailed iteration step is described below:

(i) Initialize the material parameters based on the initial condition and then determine the total stiffness matrix **K** and the inverse matrix **K**^−1^. The initial voltage *U*^1^ is $\sqrt{U_{0}^{2}/N^{U}}$ so that **P**^1^ can be calculated. According to these metrics, the temperature at the first iterative step **T**^1^ is obtained using Equation (22).
(ii) Update the material parameters according to the temperature from the previous iterative step. The applied voltage *U^n^* should also be renewed as *nU*^2^/*N*^U^ at the *n*th iterative step. After that, the matrixes $K(T^{n-1})$, $K^{-1}(T^{n-1})$, and vector $P^{n}(T^{n-1})$ are obtained. Thus, the transition temperature **T***^t^* can be calculated by Equation (23) to avoid the computational error accumulation.
  (23) $$T^{t}=K^{-1}(T^{n-1})(P^{n}(T^{n-1})-K(T^{n-1})*T^{n-1})+T^{n-1}$$
(iii) Predict and revise the transition temperature by
  (24) $$T^{\theta }=(1-\theta )T^{n}+\theta T^{t},\;\theta =0.9.$$
(iv) Re-update the material parameters using the **T***^θ^*. Further, the matrixes $K(T^{\theta })$**,** $K^{-1}(T^{\theta })$, and vector $P^{n}(T^{\theta })$ can be renewed again. Finally, the temperature **T***^n^* is obtained at the *n*th iterative step by
  (25) $$T^{n}=K^{-1}(T^{\theta })(P^{n}(T^{\theta })-P^{n-1}(T^{n-1}))+T^{n-1}.$$
(v) Termination condition judgment: If the maximum number of iterations is reached or other termination conditions are satisfied, the iteration is stopped. Otherwise, go back to step (ii).

## 4. Thermomechanical Analysis

**Figure 3:**
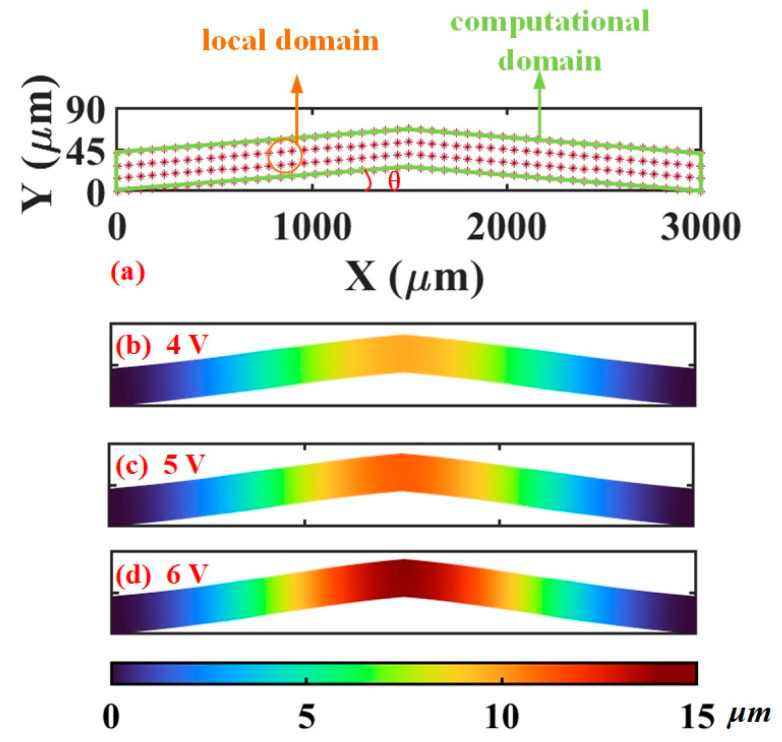
Two-dimensional computational model and displacement distribution of the actuator in the thermoelastic analysis. (**a**) Computational model. (**b**) Displacement at 4 V. (**c**) Displacement at 5 V. (**d**) Displacement at 6 V.

For the 2D model of the actuator shown in Figure 3, the steady-state thermoelastic equation is presented as

(26) $$\left\{\begin{array}{c} \begin{array}{l} \frac{E}{1-\mu^{2}}(\frac{\partial^{2}u}{\partial x^{2}}+\frac{1-\mu }{2}\frac{\partial^{2}u}{\partial y^{2}}+\frac{1+\mu }{2}\frac{\partial^{2}v}{\partial x\partial y}\ldots \\ -\frac{\partial \varepsilon_{x}^{t}}{\partial x}-\mu \frac{\partial \varepsilon_{y}^{t}}{\partial x})=0 \end{array}\\ \begin{array}{l} \frac{E}{1-\mu^{2}}(\frac{\partial^{2}v}{\partial y^{2}}+\frac{1-\mu }{2}\frac{\partial^{2}v}{\partial x^{2}}+\frac{1+\mu }{2}\frac{\partial^{2}u}{\partial x\partial y}\ldots \\ -\mu \frac{\partial \varepsilon_{x}^{t}}{\partial y}-\frac{\partial \varepsilon_{y}^{t}}{\partial y})=0 \end{array} \end{array}\right.$$

where *E* and *μ* denote the young’s modulus and passion ratio; *u* and *v* are the displacement in the *x* and *y*-direction; and *ε_x_^t^* represents the thermal strain in the X-direction. The natural boundary condition is

(27) $$\left\{\begin{array}{c} \begin{array}{l} \frac{E}{1-\mu^{2}}(n_{x}\frac{\partial u}{\partial x}+\mu n_{x}\frac{\partial v}{\partial y}+\frac{1-\mu }{2}n_{y}(\frac{\partial u}{\partial y}+\frac{\partial v}{\partial x})\ldots \\ +n_{x}(\varepsilon_{x}^{t}+\mu \varepsilon_{y}^{t}))=0 \end{array}\\ \begin{array}{l} \frac{E}{1-\mu^{2}}(n_{y}\frac{\partial v}{\partial y}+\mu n_{y}\frac{\partial u}{\partial x}+\frac{1-\mu }{2}n_{x}(\frac{\partial u}{\partial y}+\frac{\partial v}{\partial x})\ldots \\ +n_{y}(\mu \varepsilon_{x}^{t}+\varepsilon_{y}^{t}))=0 \end{array} \end{array}\right.$$

where *n_x_* and *n_y_* are components of the normal vector in the *x* and *y*-direction. The Dirichlet boundary condition at both ends of the actuator is

(28) u(0,y)=v(0,y)=u(2Lcosθ,y)=v(2Lcosθ,y)=0.

Based on the GFDM, the vector **x** and **a** in Equation (14) should be as follows.

(29) $$\begin{array}{l} x=(u_{I,x},v_{I,x},u_{I,y},v_{I,y},u_{I,xx},v_{I,xx},u_{I,yy},\ldots \\ v_{I,yy},u_{I,xy},v_{I,xy}{)}^{T} \end{array}$$

(30) $$a={\left(u_{I},v_{I},u_{1},v_{1},\ldots u_{j},v_{j},\ldots u_{N^{l}},v_{N^{l}}\right)}^{T}$$

For the discretization point (*x_I_*, *y_I_*), it should satisfy the standard form of Equation (31).

(31) $$K^{I}a=p^{I}$$

The values in the matrix **K***^I^* and *p^I^* are dependent on the location of the discretization point (*x_I_*, *y_I_*).

Case I: If the point (*x_I_*, *y_I_*) is the interior node of the computational domain, Equation (26) is adopted to obtain **K***^I^* and *p^I^*. The matrix **K***^I^* is with the 2 × (2*N^l^* + 2).
  (32) $$\left\{\begin{array}{l} K_{1,2*j-1}^{I}=\frac{E}{1-\mu^{2}}(N_{3,j}+\frac{1-\mu }{2}N_{4,j})\\ K_{1,2*j}^{I}=\frac{E}{2(1-\mu )}N_{5,j} \end{array}\right.$$
  (33) $$\left\{\begin{array}{l} K_{2,2*j-1}^{I}=\frac{E}{2(1-\mu )}N_{5,j}\\ K_{2,2*j}^{I}=\frac{E}{1-\mu^{2}}(N_{3,j}+\frac{1-\mu }{2}N_{4,j})\; \end{array}\right.$$
  and
  (34) $$\left\{\begin{array}{c} p_{1}^{I}=\varepsilon_{Ix,x}^{t}+\mu \varepsilon_{Iy,x}^{t}\\ p_{2}^{I}=\mu \varepsilon_{Ix,y}^{t}+\varepsilon_{Iy,y}^{t} \end{array}\right.$$
  where $\varepsilon_{Ix,x}^{t}$ and $\varepsilon_{Iy,x}^{t}$ are the first derivative of the *x*-direction and *y*-direction thermal strain at the point (*x_I_*, *y_I_*) versus *x*, respectively. Based on the temperature distribution, their value can be calculated via finite difference. $\varepsilon_{Ix,y}^{t}$ and $\varepsilon_{Iy,y}^{t}$ can both be regarded as 0 in light of the 1D model established in the electrothermal analysis.
Case II: If the point (*x_I_*, *y_I_*) is at the natural boundary, Equation (27) is adopted to obtain **K***^I^* and *p^I^*.
  (35) $$\left\{\begin{array}{l} K_{1,2*j-1}^{I}=\frac{E}{1-\mu^{2}}(n_{I_{x}}N_{1,j}+\frac{1-\mu }{2}n_{I_{y}}N_{2,j})\\ K_{1,2*j}^{I}=\frac{E}{1-\mu^{2}}(\mu n_{I_{x}}N_{2,j}+\frac{1-\mu }{2}n_{I_{y}}N_{1,j}) \end{array}\right.$$
  (36) $$\left\{\begin{array}{l} K_{2,2*j-1}^{I}=\frac{E}{1-\mu^{2}}(\mu n_{I_{y}}N_{1,j}+\frac{1-\mu }{2}n_{I_{x}}N_{2,j})\;\\ K_{2,2*j}^{I}=\frac{E}{1-\mu^{2}}(n_{I_{y}}N_{2,j}+\frac{1-\mu }{2}n_{I_{x}}N_{1,j}) \end{array}\right.$$
  and
  (37) $$\left\{\begin{array}{c} p_{1}^{I}=n_{Ix}(\varepsilon_{Ix}^{t}+\mu \varepsilon_{Iy}^{t})\\ p_{2}^{I}=n_{Iy}(\mu \varepsilon_{Ix}^{t}+\varepsilon_{Iy}^{t}) \end{array}\right.$$
  where *n_Ix_* and *n_Iy_* are components of the normal vector in the *x* and *y*-direction at the point (*x_I_*, *y_I_*).
Case III: Otherwise, the point (*x_I_*, *y_I_*) is at the Dirichlet boundary. Matrix **K***^I^* and *p^I^*, based on the Equation (28), are derived as follows:
  (38) $$K_{1,1}^{I}=K_{2,2}^{I}=1$$
  and
  (39) $$p_{1}^{I}=p_{2}^{I}=0,$$
  where other elements in matrix **K***^I^* are 0. Finally, the Equations (32)–(39) need to be assembled into the total matrix equation similar to (22). In this total matrix equation, **K** and **P** are with *N* × *N* and *N* × 1, respectively. For the element $K_{1,2*j-1}^{I}$, *I* is the global index of the point (*x_I_*, *y_I_*) and *j* is the local index of the point (*x_j_*, *y_j_*). *J* is the global index of the *j*. Thus, the element $K_{1,2*j-1}^{I}$ should be placed in the (2*I* − 1)^th^ row and (2*J* − 1)^th^ column of the total stiffness matrix **K**. The element $K_{2,2*j-1}^{I}$ is in the (2*I*)^th^ row and (2*J* − 1)^th^ column. The $p_{1}^{I}$ and $p_{2}^{I}$ are, respectively, located in the (2*I* − 1)^th^ and (2*I*)^th^ row of the **P**. The direct iteration method can be used to solve the total matrix equation from this analysis.

## 5. Numerical Results and Discussions

### 5.1. Thermal Prediction

**Figure 4:**
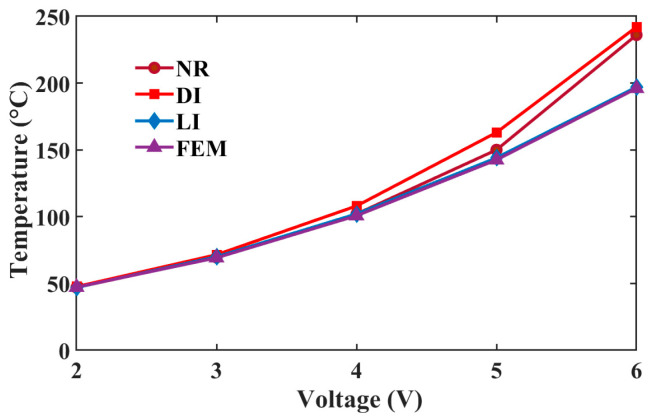
Middle temperatures of the actuator obtained by these three methods and FEM at different voltages.

As shown in Figure 2, a uniform distribution of the points is adopted in the computational domain. When combining with the GFDM and incremental load method, we capture the temperature profiles of the actuator at the steady state under different voltages. As shown in the Figure 2b–d, the temperature always arises from the two ends to the middle, where a symmetrical temperature distribution of the actuator can be observed. When a voltage of 6 V is loaded, the maximum temperature of about 195 °C lies in the central area. To validate the necessity for the incremental load method (IL), three methods, including the direct iteration (DI) and Newton–Raphson (NR), are used to solve the total matrix Equation (23). The middle temperatures obtained by these three methods and FEM at different voltages are presented in Figure 4. Compared with the result from FEM, the IL performs best in solving Equation (23). The maximum difference between them is merely about 1 °C when the actuator is loaded with 6 V. Hence, the IL can be used for the GFDM to solve the equation. In contrast, the temperature difference of the NR and DI increases with the growth of the voltage. At 6 V, their temperature differences are about 40 and 46 °C, respectively. To explain this, for the Equation (23), the thermal conductivity rises with the temperature growing. But the opposite trend occurs on the electrical resistivity. These two parameters generate the high nonlinearity of the equation and thus have a great influence on the temperature. The combination of the NR or DI with the GFDM struggles to obtain the precise solution of the equation.

**Figure 5:**
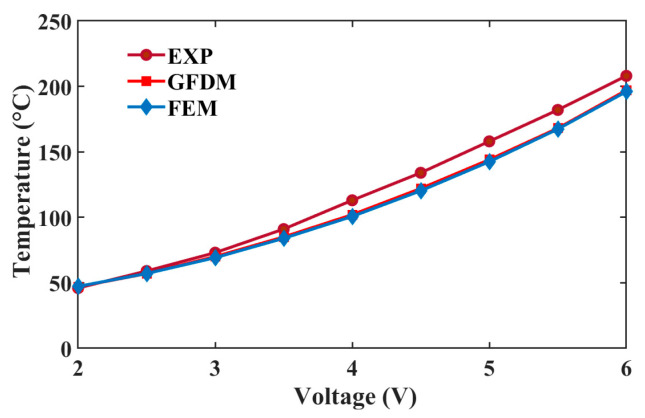
Middle temperatures comparison between the experiment, GFDM, and FEM from 2–6 V.

An infrared thermal microscope system was employed to capture the temperature profile of the actuator in experimental setup [25,26]. According to the infrared thermal image, the middle temperatures under different voltages are estimated and compared with the numerical result. As shown in Figure 5, the temperature from the GFDM agrees well with that from the FEM and the measured one. The maximum error between the GFDM and experiment is about 13 °C. The inescapable error mainly arises from Equation (15), in which the inaccurate material parameters or heat loss may be adopted. The GFDM contributes less to the error because the difference is only about 1 °C between the GFDM and FEM. That is to say, the GFDM can perfectly replace the FEM to predict the temperature in this analysis. To some degree, we need to modify the computational model to depict the thermal behavior precisely.

**Figure 6:**
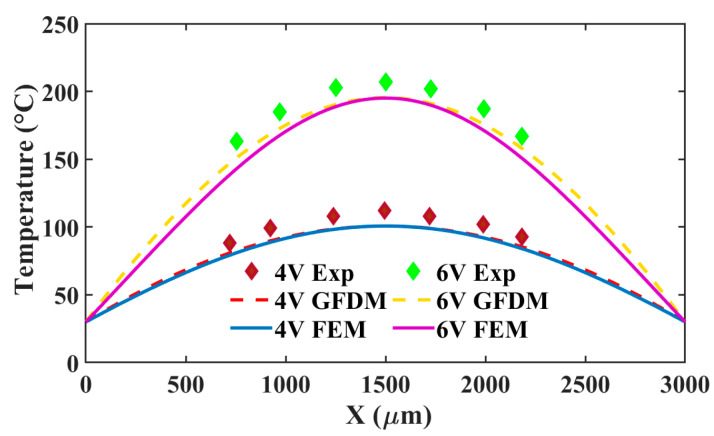
Comparison of the temperature distribution between the experiment, GFDM, and FEM at 4 and 6 V.

Limited by the size of the view field in the microscope system, the temperatures of some sample points near the middle are extracted from the infrared thermal images. As illustrated in Figure 6, a comparison of temperature distribution between experiments, GFDM, and FEM is made. Likewise, the temperature curves from the GFDM both agrees better with the curves from FEM than with experimental ones when voltages of 4 V and 6 V are applied, respectively. The maximum difference between GFDM and FEM is probably 8 °C in around one/three-quarters of the actuator under 6 V. In fact, this can just be ignored in the analysis. However, according to the experimental temperature distribution at 6 V, the temperature error gradually increases from the middle to both ends of the actuator. As estimated from 6 V in the figure, its maximum error can be up to 19 °C. Actually, the Dirichlet boundary condition used in this paper is less than perfect and it has great effect on the error. Thus, the condition requires to be amended. To do this, we may give a precise data-driven boundary condition, including the temperature derivative term, if enough experiment data can be supplied in the future.

Table 1 lists the computational stability and efficiency (CPU time) on the thermal field via GFDM simulations with different nodes when the actuator is applied with 6 V. Based on the time cost and accuracy of the calculation, the number of 101 nodes is adopted in the discretization of the 1D model. To explore the calculation stability of the incremental load method, the computational stability and efficiency (CPU time) on the thermal field by GFDM simulations with different iterative times at 6 V is listed in Table 2. The result shows that the maximum temperature of the actuator tends to converge when the iterative time *N^U^* is larger than 100.

### 5.2. Mechanical Prediction

**Figure 7:**
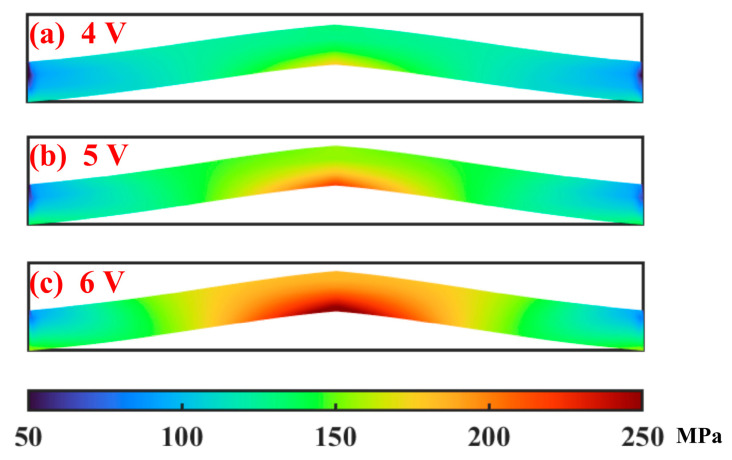
Von Mises stress distribution of the actuator.

Figure 3 shows the point distribution of the 2D computational domain of the actuator. When employing the GFDM, we capture the displacement profiles of the actuator at 4–6 V. As shown in Figure 3, the displacement always arises from the two ends to the middle, where a symmetrical displacement distribution of the actuator can also be observed. When a voltage of 6 V is loaded, the maximum displacement of about 14 μm lies in the area where the highest temperature exists. Further, the plane Von Mises stress *σ*_V_ [27] can be calculated via the following equation:

(40) $$\sigma_{V}=\sqrt{\sigma_{x}^{2}+\sigma_{y}^{2}-\sigma_{x}\sigma_{y}+3\tau_{xy}^{2}},$$

where *σ_x_* and *σ_y_* denote normal stress in *x* and *y* direction, respectively; and *τ_xy_* is the shear stress. Combined with the displacement and Equation (26), these three stresses can be obtained. Figure 7 gives the Von Mises stress distribution of the actuator at 4–6 V. Because of the structure shape of the actuator, the material flows from the bottom to the top when the deformation occurs. It is easier for the material in the top to flow and deform. The stress in the bottom area can be higher than that in the top area. Meanwhile, the material flows from the end to the middle because of the limit on the displacement in both ends. Hence, the peak Von Mises stress should be located in the bottom of the middle area of the actuator, which can be validated in the figure. When a voltage of 6 V is loaded, the peak Von Mises stress is about 247 MPa.

**Figure 8:**
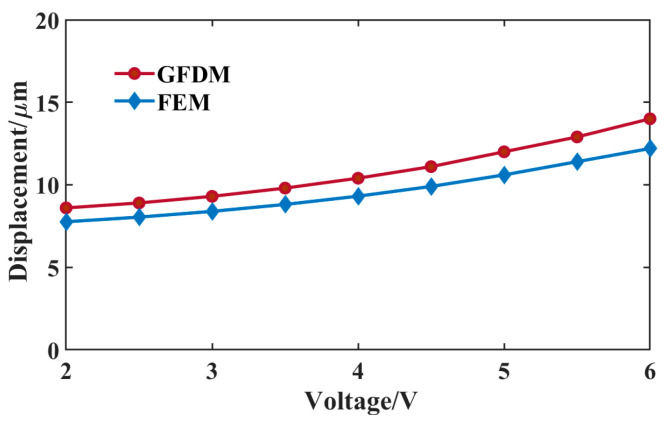
Maximum displacement of the actuator via the GFDM and FEM loaded with different voltages.

Under different voltages, the displacements in the middle area of the actuator, calculated by the GFDM and FEM, respectively, are presented in Figure 8. In the figure, the displacement curve for the GFDM is a little larger than that for the FEM. The maximum error of the displacement is about 1.8 μm when 6 V is applied. This error partly results from the temperature difference between GFDM and FEM, which is found in Figure 6. Table 3 lists the computational stability and efficiency (CPU time) on the mechanical field by GFDM simulations with different nodes at 6 V. From the table, the maximum displacement will converge with the number of growing nodes. Because of the use of the direct iteration method, its computational efficiency is far higher than that in thermal prediction when dealing with the same amount of nodes. Given its good performance in the mechanical prediction, the GFDM can be employed to solve the thermomechanical model in the actuator.

## 6. Conclusions

This paper provides a successful application of the GFDM to solve the electric, thermal, and mechanical field coupling problems of the V-shaped micro-actuator. For the electrothermal part, benefiting from the IL iterative strategy, the difference in the highest temperature at the middle of the actuator is no more than 1 °C and 13 °C when compared with the FEM and experiment results at 6 V, respectively. According to the temperature distribution at 6 V, the maximum temperature errors are 8 °C and 19 °C, respectively. The simulation and experimental results verify the combination of the GFDM with the IL strategy, used to handle the highly nonlinearity in the electrothermal analysis. For the thermomechanical part, with the implementation of the GFDM, the mechanical characteristics are also perfectly predicted. The maximum displacement error is about 1.8 μm between the GFDM and FEM, when a voltage of 6 V is loaded. Therefore, these results prove that the GFDM-based scheme is an efficient, stable, and precise numerical method for multi-physics field problems.

In addition, given the increasing temperature error from the middle to both ends in this study, a modification of the boundary condition by the experiment data and physics-informed neutral network (PINN) may be considered in our future.

## Figures and Tables

**Table 1:** Computational stability and efficiency (CPU time) on the thermal field by GFDM simulations with different nodes at 6 V.

*N*	11	101	501	1001
Max temperature (°C)	189.1	195.3	195.2	195.2
CPU time (s)	0.04	0.39	2.64	8.29

**Table 2:** Computational stability and efficiency (CPU time) on the thermal field by GFDM simulations with different iterative times at 6 V.

*N^U^*	10	50	100	1001
Max temperature (°C)	216.1	197.8	195.3	195.1
CPU time (s)	0.06	0.22	0.39	1.64

**Table 3:** Computational stability and efficiency (CPU time) on the mechanical field via GFDM simulations with different nodes at 6 V.

*N*	201	505	1206	2406
Max displacement (μm)	11.4	13.9	14.0	14.0
CPU time (s)	0.05	0.06	0.17	0.84

## Data Availability

The original contributions presented in the study are included in the article, further inquiries can be directed to the corresponding author.

## References

[1] An L., Ogura I., Ashida K., Yabuno H. (2024). Mechanism of mechanical nanolithography using self-excitation microcantilever. Nonlinear Dyn..

[2] Jalili H., Salarieh H., Vossoughi G. (2017). Study of a piezo-electric actuated vibratory micro-robot in stick-slip mode and investigating the design parameters. Nonlinear Dyn..

[3] Gao X., Deng J., Zhang S., Li J., Liu Y. (2022). A compact 2-dof micro/nano manipulator using single miniature piezoelectric tube actuator. IEEE Trans. Ind. Electron..

[4] Kong X., Cao Y., Zhu H., Nie W., Xi Z. (2023). A self-latching MEMS optical interrupter with status monitoring for laser initiation system. IEEE Trans. Electron Devices.

[5] Dai J., Feng C., Xie J., Gao M., Zhen T. (2023). Design and control of an analog optical switch based on the coupling of an electrothermal actuator and a mass–spring system. IEEE/ASME Trans. Mechatron..

[6] Ghanbari M., Rezazadeh G., Moloudpour-Tolkani V., Sheikhlou M. (2023). Dynamic analysis of a novel wide-tunable microbeam resonator with a sliding free-of-charge electrode. Nonlinear Dyn..

[7] Benedetti K.C.B., Gonçalves P.B. (2022). Nonlinear response of an imperfect microcantilever static and dynamically actuated considering uncertainties and noise. Nonlinear Dyn..

[8] Ma M., Ekinci K.L. (2023). Electrothermal actuation of NEMS resonators: Modeling and experimental validation. J. Appl. Phys..

[9] Yunas J., Mulyanti B., Hamidah I., Said M.M., Pawinanto R.E., Ali W.A.F.W., Subandi A., Hamzah A.A., Latif R., Majlis B.Y. (2020). Polymer-based MEMS electromagnetic actuator for biomedical application: A review. Polymers.

[10] Ouakad H.M., Krzysztof Kamil U.R. (2022). On the snap-through buckling analysis of electrostatic shallow arch micro-actuator via meshless galerkin decomposition technique. Eng. Anal. Bound. Elem..

[11] Khorasani V.S., Żur K.K., Kim J., Reddy J. (2022). On the dynamics and stability of size-dependent symmetric FGM plates with electro-elastic coupling using meshless local Petrov-Galerkin method. Compos. Struct..

[12] Chen H., Sun X., Chen M., Kong X. (2025). Dynamic response of a MEMS electrothremal actuator by the local radial point interpolation method. Nonlinear Dyn..

[13] Nguyen B.H.H., Torri G.B., Rochus V. (2024). Physics-informed neural networks with data-driven in modeling and characterizing piezoelectric micro-bender. J. Micromech. Microeng..

[14] Xia H., Gu Y. (2021). The generalized finite difference method for electroelastic analysis of 2D piezoelectric structures. Eng. Anal. Bound. Elem..

[15] Shao Y., Duan Q., Chen R. (2024). Adaptive meshfree method for fourth-order phase-field model of fracture using consistent integration schemes. Comput. Mater. Sci..

[16] Demkowicz L.F., Gopalakrishnan J. An overview of the discontinuous Petrov Galerkin method. Proceedings of the Recent Developments in Discontinuous Galerkin Finite Element Methods for Partial Differential Equations: 2012 John H Barrett Memorial Lectures.

[17] Cuomo S., Di Cola V.S., Giampaolo F., Rozza G., Raissi M., Piccialli F. (2022). Scientific machine learning through physics–informed neural networks: Where we are and what’s next. J. Sci. Comput..

[18] Guo R., Sui F., Yue W., Wang Z., Pala S., Li K., Xu R., Lin L. (2022). Deep learning for non-parameterized MEMS structural design. Microsyst. Nanoeng..

[19] Xu B., Zhang R., Yang K., Yu G., Chen Y. (2023). Application of generalized finite difference method for elastoplastic torsion analysis of prismatic bars. Eng. Anal. Bound. Elem..

[20] Sun W., Qu W., Gu Y., Zhao S. (2023). Meshless generalized finite difference method for two-and three-dimensional transient elastodynamic analysis. Eng. Anal. Bound. Elem..

[21] Zhao Q., Fan C.-M., Wang F., Qu W. (2020). Topology optimization of steady-state heat conduction structures using meshless generalized finite difference method. Eng. Anal. Bound. Elem..

[22] Ju B., Yu B., Zhou Z. (2024). A generalized finite difference method for 2D dynamic crack analysis. Results Appl. Math..

[23] Chen H., Wang X.-J., Wang J., Xi Z.-W. (2020). Analysis of the dynamic behavior of a V-shaped electrothermal microactuator. J. Micromech. Microeng..

[24] Zhu H., Cao Y., Ma W., Kong X., Lei S., Lu H., Nie W., Xi Z. (2024). Dynamic modeling of a mems electro-thermal actuator considering micro-scale heat transfer with end effectors. J. Microelectromech..

[25] Cao Y., Liu H., Chen H., Zhu H., Nie W., Xi Z. (2021). Dynamic thermal characterization of MEMS electrothermal actuators in air. IEEE Sens. J..

[26] Kong X., Cao Y., Zhu H., Wang H., Lu J., Xu X., Nie W., Xi Z. (2024). Experimental and numerical study of MEMS electrothermal actuators: Comparing dynamic behavior and heat transfer process in vacuum and non-vacuum environments. Vacuum.

[27] Asgari M., Kouchakzadeh M.A. (2019). An equivalent von Mises stress and corresponding equivalent plastic strain for elastic–plastic ordinary peridynamics. Meccanica.

